# Bioactivity and Digestibility of Microalgae *Tetraselmis* sp. and *Nannochloropsis* sp. as Basis of Their Potential as Novel Functional Foods

**DOI:** 10.3390/nu15020477

**Published:** 2023-01-16

**Authors:** Samuel Paterson, Pilar Gómez-Cortés, Miguel Angel de la Fuente, Blanca Hernández-Ledesma

**Affiliations:** Department of Bioactivity and Food Analysis, Institute of Food Science Research (CIAL, CSIC-UAM, CEI UAM+CSIC), Nicolás Cabrera 9, 28049 Madrid, Spain

**Keywords:** microalgae, nutritional value, bioactivity, digestibility, novel functional foods

## Abstract

It is estimated that by 2050, the world’s population will exceed 10 billion people, which will lead to a deterioration in global food security. To avoid aggravating this problem, FAO and WHO have recommended dietary changes to reduce the intake of animal calories and increase the consumption of sustainable, nutrient-rich, and calorie-efficient products. Moreover, due to the worldwide rising incidence of non-communicable diseases and the demonstrated impact of diet on the risk of these disorders, the current established food pattern is focused on the consumption of foods that have functionality for health. Among promising sources of functional foods, microalgae are gaining worldwide attention because of their richness in high-value compounds with potential health benefits. However, despite the great opportunities to exploit microalgae in functional food industry, their use remains limited by challenges related to species diversity and variations in cultivation factors, changes in functional composition during extraction procedures, and limited evidence on the safety and bioavailability of microalgae bioactives. The aim of this review is to provide an updated and comprehensive discussion on the nutritional value, biological effects, and digestibility of two microalgae genera, *Tetraselmis* and *Nannochloropsis*, as basis of their potential as ingredients for the development of functional foods.

## 1. Introduction

Microalgae are single-celled, prokaryotic, and eukaryotic primary photosynthetic microorganisms with diverse taxonomical and phylogenetical characteristics. Their distinguished ability to produce biomass from CO_2_, solar energy and elemental nutrients combined with their adaptation capacity to extreme surroundings have led to their presence in a myriad of environmental media throughout evolution [1,2]. Microalgae are classified on the basis of several features, such as the nature of the photosynthetic storage products, the organization of photosynthetic membranes, and the pigmentation, among others. The term microalgae includes different groups of eukaryotic organisms such as chlorophylls (green algae), bacillaryiophytes (diatoms), dinophytes (dinoflagellates), euglenophytes, prymne-siophytes (cocolitophorides) and prokaryotes, such ascaryophytes [2].

*Tetraselmis* sp. are green unicellular flagellates that belong to the family of *Chlorodendraceae* within the class of *Chlorodendrophyceae*, with almost spherical, slightly flattened cells with an invagination at the anterior end from which four equal flagella arise in two opposite pairs. Nowadays, numerous marine and freshwater species of this microalgae are known and some even occur in plankton [3]. *Tetraselmis* sp. have been used as a laboratory model strain to study the effect of diverse conditions such as saline or high nitrogen environments on their growth, survival and adaptation [4]. Moreover, these species are frequently used in aquaculture due to their high nutritional value in terms of proteins and fatty acids, and their content in other biological compounds [5]. Different strains of the *Tetraselmis marina* species have also been considered an innovative, cost-effective, efficient and sustainable method for the removal of toxic substances such as heavy metals from wastewater and aquatic ecosystems [6]. Recently, *Tetraselmis chuii* has been approved by the European Food Safety Authority as a novel food [7]. Moreover, the Scientific Committee of the Spanish Agency for Food Safety and Nutrition has declared that there is no indication that consumption of the *T. chuii* microalgae as a condiment can produce adverse effects on health [8].

*Nannochloropsis* sp. are unicellular, planktonic, with either 2–4 μm diameter subspherical or 3–4×1.5 μm cylindrical cells [9]. They belong to the family of *Monodopsidaceae* within the class of *Eustigmatophyceae*. *Nannochloropsis* sp. grow mainly in marine environments, and they normally contain a yellow-green chloroplast with the main pigments being chlorophyll A and xanthophylls [10]. Currently, seven *Nannochloropsis* sp. are recognized, six detected in marine environments (*N. salina, N. australis, N. granulata, N. oceanica, N. oculata,* and *N. gaditana*), and *N. limnetica,* which can be located in both fresh and brackish water [9]. These species of *Nannochloropsis* have become very popular for their use in animal feeding, aquaculture, and water waste treatment. In addition, their lipid production for biofuels and the production of long-chain polyunsaturated fatty acids (PUFAs), especially eicosapentaenoic acid (EPA, C20:5 n-3), are some of their main areas of interest to researchers [11,12]. Nevertheless, other different applications such as the development of natural cosmetics and cosmeceuticals are being currently explored [13].

**Figure 1:**
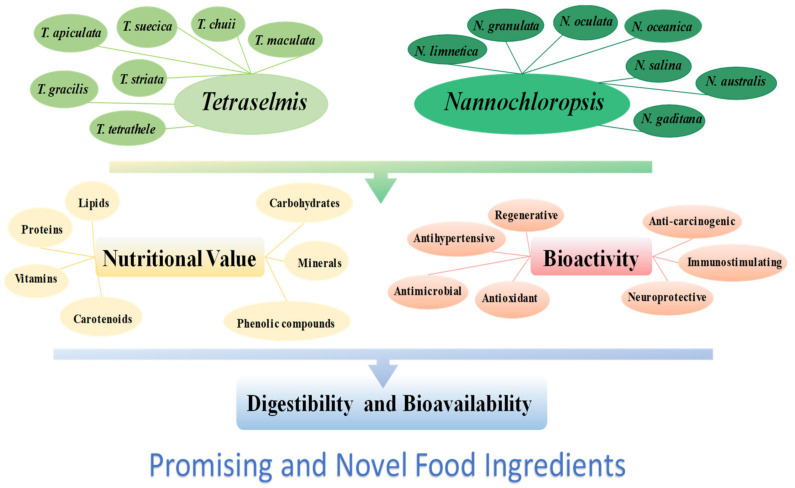
Overview of the nutritional value and bioactivities demonstrated for microalgae *Tetraselmis* sp. and *Nannochloropsis* sp. species.

Overall, *Tetraselmis* sp. and *Nannochloropsis* sp. microalgae provide environmental benefits, have a rich composition in nutrients and industrial applications as remarkable properties. However, the full applications and potential biotechnological roles of these microalgae in the food sector will directly depend on the bioactivity, digestibility, and bioavailability of their nutritional and bioactive compounds. Information about these genera is very abundant in the literature but it is rather scattered. This review aims to compile and update the composition, nutritional value, biological activity, and digestibility of *Tetraselmis* sp. and *Nannochloropsis* sp. microalgae biomass and derived compounds (Figure 1).

## 2. Nutritional Value of *Tetraselmis* and *Nannochloropsis* sp. Microalgae

Microalgae are considered rich sources of macro- and micronutrients such as lipids, proteins, carbohydrates, minerals, and vitamins, among others. The biochemical composition of *Nannochloropsis* and *Tetraselmis* biomass show quantitative variations depending on the microalga strain as well as their growing environmental conditions. It is also remarkable that most of the existing composition data in literature come from microalgae cultivated on a laboratory scale, frequently different from industrial cultivation, thus affecting the nutrient profile.

### 2.1. Proteins and Amino Acids

Overall, *Nannochloropis* and *Tetraselmis* genera can be considered as a good source of proteins with desirable amino acid profile. Proteins in *Tetraselmis* range from 13% to 48% in dry weight (DW) biomass basis, although most species have protein levels of approximately 30%, mainly depending on the growing environmental factors [14,15,16]. The introduction of *Nannochloropsis* as a novel food is also justified by the high protein of appreciable nutritional value [17]. Protein content and its bioavailability are also affected by numerous biotic and abiotic factors and genomic variation among species. Values reported in the bibliography ranged from 19 to 24% for *N. oculata* [18] to 44.9% for *N. gaditana* [19]. Levasseur et al. [15] also compiled intermediates percentages for this genus.

The amino acid profile is important to assess the nutritional quality of foods. The quality of proteins can be determined specifically by the presence of essential amino acids (EAA). The amino acid composition of *Nannochloropsis* and *Tetraselmis* is listed in Table 1 together to reference values for EAA recommended by the Food and Agriculture Organization (FAO). Comparison of EAA profile of microalgae proteins with FAO reference pattern indicated that, overall, the EAA content of these microalgae is close to reference values that are indicative of good protein quality. However, methionine content is lower than stated by FAO. Microalgae also have a rich and varied composition of non-essential amino acids (Table 1), among which the most abundant are aspartate and glutamate (*Nannochloropsis*) and arginine (*Tetraselmis*). Other studies in *T. chuii* [20,21] confirm the quantitative importance of aspartic and glutamic acids in their proteins.

### 2.2. Lipids and Fatty Acids

The biomass of *Nannochloropsis* sp. is widely used as a source of healthy and essential lipids due to its high content in these molecules. *Nannochloropsis* has a lipid content (37 to 60% of DW) higher than that of other microalgae [23]. *Nannochloropsis* species are known to be rich in n-3 PUFAs, mainly EPA (C20:5 n-3), with contents ranging from 1.1% to 11% of its DW biomass [17]. Other dominant fatty acids are palmitic (16:0), palmitoleic (*cis*-9 16:1) and, with less abundance, oleic (*cis*-9 18:1) acids (Table 2). In contrast, the presence of lipids in species of *Tetraselmis* genus is clearly lower, between 5–10% DW [16,21,24]. The fatty acid profile is mainly composed of palmitic, oleic, linoleic (*cis*-9 *cis*-12 C18:2, n-6), and α-linolenic (*cis*-9 *cis*-12 *cis*-15 C18:3 n-3) acids. Other omega-3 PUFAs as EPA or C22:6 n-3 are less abundant (Table 2).

The lipids of microalgae can be modified under experimental conditions. Illumination, nitrogenous (N):phosphorus (P) ratio and salinity are essential factors to influence the content and profile of lipid classes and fatty acids [23,31]. Furthermore, this behavior varies differently across species when exposed to the same culturing conditions. Cultures generally tend to accumulate lipids in slow cell proliferation phases, when most of the energy is available in reserve molecules, whereas EPA and other PUFAs increase with the cell density because of their role as cytoplasmic membrane components [32]. Van Wagenen et al. [33] postulated that the amount of PUFAs and EPA are inversely proportional to temperature, as physiological adaptations aimed to maintain the cell membrane fluidity.

Microalgae synthesize fatty acids mainly for esterification into glycerol-based membrane lipids but n-3 PUFAs can also be esterified to polar lipids such as phospholipids and glycolipids, which are more bioavailable [34]. These polar lipids can be abundant in the biomass of microalgae as *Nannochloropsis* [35,36] and *Tetraselmis* [37]. The *Nannochloropsis* sp. polar lipids have well-known bioactivities, including anti-inflammatory action [38]. Kagan et al. [39] demonstrated that polar lipid rich oil from *Nannochloropsis oculata* was an effective source of EPA in humans while Rao et al. [40] observed that supplementation with an EPA polar lipid extract of *Nannochloropsis* sp. increased this omega-3 PUFA as well as reduced the cholesterol level in healthy individuals.

### 2.3. Carbohydrates

Carbohydrates are nutrients also present in microalgae, both as intracellular storage molecules and cell wall structural elements. Zanella and Vianello [17] reported a range from 6.4 to 36.2% as fraction DW biomass in a variety of species of *Nannocholoropsis*. Carbohydrate’s content can be manipulated through growth conditions. For instance, *T. suecica* was able to accumulate up to 37% under N-starved conditions, but when it was grown in nutrient replete medium, the carbohydrates content ranged from 6 to 10% [30]. With controlled growth conditions, *Tetraselmis* sp. carbohydrate contents were approximately 10% but under stressful conditions their values could exceed 50% [15].

Polysaccharide is the major form of carbohydrate in all species of microalgae. On average, in *Tetraselmis* and *Nannochloropsis*, approximately 90% of total carbohydrates are polysaccharides [41]. In *Nannochloropsis* sp., cellulose is the main component of its tough and highly recalcitrant cell wall [42]. These cell wall polysaccharides were first exploited for their rheological properties but could also be applied in the food and pharmaceuticals industries [15]. Species of *Nannochloropsis* genus may also be a source of β-glucans, glucose polymers linked by β-1,3, β-1,4 and branches of β-1,6 bonds [43]. β-glucans attract attention because of their bioactive properties and could grant a high potential to the culture of *Nannochloropsis* with commercial purposes [44].

The main intracellular polysaccharide described in *Tetraselmis* genus is starch [16], a storage macromolecule common in the vegetable word and, from a nutritional point of view, an important ingredient as source of energy. In addition, the cell wall polysaccharides of this genus have been reported to contain 3-deoxy-d-manno-oct-2-ulosonic acid, a molecule with pharmacological properties and a high economic value. This monosaccharide has been identified in *Tetraselmis striata*, *T. tetrathele* and *T. suecica* [45,46].

The sugar composition of the polysaccharides from microalgae differs between species and the variations could contribute to differences in the nutritional value, since animals digest polysaccharides of different composition at different rates. In *N. oculate,* glucose, the preponderant sugar content (68.2%) is followed by that of fucose, galactose, mannose, rhamnose, ribose and xylose in percentages below 10% [41]. A similar sugar pattern was reported by Templeton et al. [47] in *Nannochloropsis* sp. In *T. chuii* and *T. suecica*, glucose is also the principal sugar (>74%) while galactose, ribose, mannose, rhamnose, and arabinose were detected in lower percentages [41]. Pereira et al. [16] showed that *Tetraselmis* sp. CTP4 biomass is composed mainly of glucose too, including galactose, mannose arabinose and xylose contained in lower proportions.

### 2.4. Mineral Elements

Mineral data of *Nannochloropsis* and *Tetraselmis* genera are provided in Table 3. Major elements reported in these studies were Ca, Mg, K, Na, P and S. Minor and trace element compositions included Cu, Fe, Mn, Zn, and I. Furthermore, very low amounts of Se, Ni and Mo were also reported by some authors [21,22,48]. In *Nannochloropsis*, the richest elements detected were Na [22,42], K [48] and Ca [32], whereas Na [42], K [16], Ca [48] and Mg [21] were the richest detected in *Tetraselmis*. Iron was clearly the most abundant trace mineral in all these microalgae, with a concentration ranging from 30 to 177 mg/100 g of DW biomass.

There were very large differences in mineral elements concentrations between the species obtained from different studies (Table 3). Furthermore, the concentrations of minerals showed big differences, even within the same species. For instance, in *T. chuii*, the amount of Ca reported by Sandgruber et al. [21] was 15 times lower than that of *T. chuii* powder according to Tibbetts et al. [48]. This variability was greater for most of the trace elements contents and might result from different cultivation conditions, particularly the composition of the growing medium. Moreover, the concentration of the elements depends on the harvest of the microalgae in different growing stations in which elements were accumulated [21].

### 2.5. Vitamins

Microalgae can synthesize and accumulate a wide range of vitamins and could be a potential source of these bioactive compounds compared to some well-known and traditional vegetables [15,49,50]. In Table 4, integrated state-of-the-art data on the vitamins A, C, D, E, K and B complex corresponding to *Nannochloropsis* and *Tetraselmis* genera can be found.

*T. suecica* presents the highest content of vitamin A and it seems to be rich in vitamins B1, B2, B3 and D2 [51,52]. Moreover, tocopherols values are especially high in this specie with concentrations up to 1.980 mg/100 g DW [53]. In *Nannochloropsis* genus, because of limited data, levels are still difficult to assess. Vitamin A was not detected or it was recorded in very low amount [54]. In contrast, *N. gaditana* powders have been shown to be a good source of vitamin B9 [55]. Its content (2.080 μg/100 g DW) represents approximately a quarter of the recommended daily intake (400 μg) for a consumption of 5 g of microalgal powder [15].

**Table 4:** Vitamin content in *Nannochloropsis* sp. and *Tetraselmis* sp. microalgae biomass.

Vitamin	Content (mg/100 g)											
	*T.* *suecica* ^1^	*T.* *suecica* ^2^	*T.* *suecica* ^3^	*N.* sp. CS-246 ^3^	*T.* sp. CS362 ^4^	*T.* sp. CTP4 ^5^	*N.* sp.^4^	*N.* *oculata* ^6^	*N. limnetica* ^7^	*N. salina* ^7^	*N. gaditana* ^8^	*N. oceanica* ^9^
A *	29625	428000		<25	220	<4						
B1	3.23	62.70		5.10-7.00	10.90	0.18						
B2	1.91	4.20		2.50-6.20	2.60	0.53					2.21	
B3	8.93	141				7.98					11	
B5	3.77					0.65						
B6	0.28	15.50		0.36-0.95	0.58	6.90						
B7	0.08			0.10-0.11	0.13							
B9 *	300			1700-2600	2000	0.02					2080	
B12 *	50.00	900		85-170	195	7.80					25	
C	19.10	49.80		100-320	300	79.20						
D2 *		1400		45.0	<35							
D3 *				35.0	<35							1–48
E	42.18	632	1980	18.0-35.0	7.00	20.28	210	48-233	2.12	4.41		
K *		2800										

* Values expressed as µg/100 g. ^1^ Fabregas and Herrero [52]; ^2^ De Roeck-Holtzhauer et al. [51]; ^3^ Santiago-Morales et al. [53]; ^4^ Brown et al. [54]; ^5^ Pereira et al. [16]; ^6^ Durmaz [56]; ^7^ Safafar et al. [57]; ^8^ Edelman et al. [55]; ^9^ Ljubic et al. [58].

As can be seen in Table 4, within individual studies, the vitamin content of different species can differ by a factor of ten or more. Even larger variations have been detected among studies [50]. For instance, the B12 content of *T. suecica* was documented by De Roeck-Holtzhauer et al. [51] to be 900 µg/100 g DW, whereas it was reported to be 50 by Fábregas and Herrero [52]. These marked differences must be attributed to variations in culture conditions, changes in the harvesting technique as well as specific analytical techniques employed by the different laboratories [54]. In this line, nutrient depletion enhanced the production of vitamins C and E in *T. suecica* [59]. Similarly, α-tocopherol accumulated along with N concentration decrease in *N. oculata*, or with the addition of N and P in *T. suecica* [50]. Among four different microalgaes, *N. oceanica* it was the only one able to produce vitamin D3 and this production was enhanced by culture irradiation with ultraviolet B [58]. Pereira et al. [16] attributed the low values of several vitamins in *Tetraselmis* sp. CTP4 to the high temperatures (>50 °C) applied in the industrial spray-drying process to produce microalgae biomass.

### 2.6. Carotenoids

Carotenoids are a diverse and widespread class of bioactive compounds produced by microalgae which play essential roles as accessory light harvesting pigments and photoprotective agents. Carotenoids are divided into carotenes, oxygen-free hydrocarbons such as α-carotene or β-carotene, and xanthophylls, oxygenated derivatives of carotenes (lutein, violaxanthin, fucoxanthin and astaxanthin, among others).

Total content of carotenoids is extremely variable. In *Nannochloropsis*, the measured levels ranged from 0.4 mg/g DW [32] to 5.3 [57]. Banskota et al. [28] documented the highest levels of carotenoids in *T. chuii* (8.5 mg/g DW), although lower values were frequently found in the literature [37,59,60] for other strains of this genus. Table 5 summarizes the carotenoid profile of *Nannochloropsis* and *Tetraselmis* genera reported in the bibliography. Up to 15 different individual molecules were compiled from various studies. β-carotene was the only carotenoid detected in all samples, whereas violaxanthin is the xantophill present in most of the studies. A higher proportion of xantophills (up to 80%) than that of carotenes was usual, with a great quantitative and qualitative variety of compounds in the first group. As can be seen in Table 5, this composition showed large variations between different strains, too. For instance, the qualitative profile of *Tetraselmis* sp. Z3 was comparable to that of *Tetraselmis* sp. C6, but the contents of every molecule were significantly higher for the latter.

The whole variability denoted in Table 5 is justified by several pieces of evidence. Basic carotenoid synthesis pathways, mainly β-carotene, are similar in most microalgae. However, since the activation of carotenogenesis is under strict genetic control [60], different species or strains can accumulate unusual molecules via specific biosynthetic routes [61]. Additionally, as profusely observed for proteins and lipids, carotenoids synthesis by microalgae occurs in response to environmental stress factors, and culture conditions severely affect its production [15,60]. For example, in *Nannocholoropsis*, carotenoids can increase under low light irradiation or penetration, due to high culture density, restriction of P and S and changes in N-source ingredients in the culture media [17].

**Table 5:** Carotenoids composition (expressed as mg/g of dry weight, DW) of *Nannochloropsis* sp. and *Tetraselmis* sp. microalgae.

Compound	Carotenoids (mg/g DW)												
	*T.* *suecica* ^1^	*T.* *suecica* ^2^	*T.* *chuii* ^2^	*T.* *chuii* ^3^	*T.* sp. Z3 ^4^	*T.* sp. C6 ^4^	*N. gaditana ^1^*	*N.* *gaditana* ^5^	*N.* sp. BR2 ^2^	*N.* *granulata* ^3^	*N.* sp. ^5^	*N. salina* ^6^	*N.* *limnetica* ^6^
Carotene													
α-carotene	0.41	0.20	0.17	1.70		0.02						0.08	
β-carotene	0.43	0.62	0.94	1.00	0.002	0.01	1.00		0.45	1.00		2.22	0.28
Lycopene				1.50									
Total Carotenes								1.15			1.16		
*Xanthophyll*													
Cantaxanthin				0.40						0.40		0.14	0.003
Zeaxanthin				0.10			0.01	0.90		0.10	0.20	0.58	0.14
Violaxanthin	0.82	1.41	0.54				3.36	4.45	1.08		4.17	1.68	1.23
Lutein	0.85	0.48	0.62	0.60	0.37	1.65		0.02					
Anteraxanthin		0.83	0.20						0.17				
Astaxanthin		2.26		0.10					0.32				
Fucoxanthin					0.05	0.08						0.01	0.18
Neoxanthin					0.12	0.79		0.01				0.05	0.42
Diadinoxanthin								1.44			1.18	0.14	
Vaucheriaxanthin												0.09	0.16
Alloxanthin												0.13	
Diatoxanthin													0.14

^1^ Di Lena et al. [60]; ^2^ Ahmed et al. [62]; ^3^ Banskota et al. [28]; ^4^ Grubišic et al. [37]; ^5^ Ryckebosch et al. [63]; ^6^ Safafar et al. [57].

### 2.7. Phenolic Compounds

Phenolic compounds are a large chemical family of metabolites including acids, alcohols, and flavonoids among other molecules, which contain a polyphenol structure consisting of six-carbon aromatic rings. *Nannochloropsis* and *Tetraselmis* genera could be a potential source of phenolic compounds (Table 6) and, as it can be observed, their concentrations are highly variable.

Although the highest levels were detected in *N. gaditana* extract with 32.0 mg gallic acid equivalent (GAE)/g DW [64] followed by *T. suecica* with 28.03 mg GAE/g DW (Haoujar et al., 2019), most of the reported concentrations were below 10 mg GAE/g DW. In this regard, it should be taken into consideration that phenolic composition is strongly influenced by microalgae species and growth conditions [57,65]. Furthermore, the extracting solvents used (water, alcohols, or organic solvents) significantly affect phenolic content [66,67].

There are few published studies regarding the identification and quantification of individual phenolic compounds in *Nannochloropsis* and *Tetraselmis* genera. Such works show that phenolic profiles may be quite different, even when comparing to microalgal species of the same genus (Table 6). For instance, the very significant differences detected by Cardoso et al. [66] between two *Tetraselmis* strains are noteworthy. Caffeic acid (53.3 mg/L) and luteolin-7-o-glucoside (9 mg/L) were the most abundant molecules in *Tetraselmis* sp. IMP3, but those compounds remained undetected in *Tetraselmis* sp. CTP4. Safafar et al. [57] only identified simple phenolic acids in *Nannochloropsis salina* and *Nannochloropsis limnetica* as gallic, 3,4-dihydroxy benzoic, caffeic, p-coumaric, salycilic, cinnamic and ferulic, the latter being the most abundant phenolic acid in both species (2.90 and 2.45 µg/g DW, respectively). In contrast, chlorogenic acid was clearly (489.5 µg/g DW) the richest phenolic acid in *Nannochloropsis* sp. [67]. Protocatechuic acid, caffeic acid, and p-coumaroyl tyrosine were detected in *T. suecica* and *N. gaditana*, with the highest content in the former [68]. For the flavonoids class, apigenin-O-rutinoside and rhamnosylhexosyl-methyl-quercetin were identified (Table 6).

**Table 6:** Total content (mg of gallic acid equivalent/g of dry weight) and identified phenolic compounds in biomass of *Nannochloropsis* and *Tetraselmis* genera.

Microalgae	Total Content	Individual Phenolic Compounds	Ref.
*Nannochloropsis* sp.	1.4	---	[65]
*Nannochloropsis* sp.	0.6	Gallic, ferulic, protocatechuic, chlorogenic, hydroxybenzoic, syringic, vanillic	[67]
*N. gaditana*	32.0	---	[64]
	22.9	Caffeic, caffeoylglucoside, protocatechuic, *p*-coumaroyl tyrosine, quercetin, rhamnosyhexosyl-methyl-quercetin, apigenin-O-rutinoside, feruloylglucaricacid	[68]
*N. granulata*	6.0–8.0	---	[48]
*N. limnetica*	5.8	Gallic, caffeic, ferulic, cinnamic, salycilic	[57]
*N. oculata*	2.0	---	[65]
	4.1	---	[69]
*N. salina*	6.8	Gallic, 3,4 dihydroxy benzoic, ferulic, *p*-coumaric, salycilic	[57]
*Tetraselmis* sp.	3.7	---	[65]
*Tetraselmis* sp.	25.5	---	[64]
*T.* sp. IMP3	2.3	Gallic, gentisic, catechin hydrate, 4-hydroxybenzaldehyde, vanillic, caffeic, epicatechin, ferulic salicylic, naringenin-7-glucoside, luteolin-7-o-glucoside, rutin, ellagic, quercetin	[66]
*T.* sp. CTP4	0.76	Gallic, *p*-hydroxybenzoic, catechin hydrate, epicatechin	[66]
*T. chuii*	8.6	---	[69]
	20.0	---	[48]
*T. suecica*	1.7	---	[65]
	28.0	Caffeic, caffeoylglucoside, protocatechuic, dimethoxyflavone, *p*-coumaroyl tyrosine, apigenin-*O*-rutinoside, rhamnosyhexosyl-methyl-quercetin	[68]

## 3. Bioactivity of *Tetraselmis* sp. and *Nannochloropsis* sp. Microalgae

In addition to their nutritional role, foods are being recognized as a source of non-nutritive bioactive compounds able to prevent or reduce the risk of non-communicable disorders, such as neurodegenerative and cardiovascular diseases, obesity and diabetes, and cancer. Although plant foods are a food category containing both nutritive and bioactive compounds, the increase in the world population, subsequent food demand and the higher consumer knowledge on the food/diet relationship have promoted and engaged the search for new sustainable food sources by the food industry. Among these new sources, microalgae are becoming a promising functional food supply. The health benefits attributed to these aquatic microorganisms are associated to the presence of both primary metabolites or nutrients (lipids, proteins, carbohydrates, and vitamins) and secondary metabolites (phenolic compounds, carotenoids, among others). Biologically active substances from microalgae are capable of exhibiting antioxidant, antibacterial, antiviral, antitumor, regenerative, antihypertensive, neuroprotective and immunostimulating effects [70]. Thus, microalgae and derived compounds have an important potential as natural and sustainable dietary supplements for the prevention, management, and treatment of chronic disorders [71]. However, although the number of microalgae species was estimated to be over 168,000 [72], only few species are currently cultivated on a large scale and just a limited number have been studied for commercial purposes. Nevertheless, the biodiversity of microalgae would allow to increase the sources of bioactive metabolites with potential therapeutic applications as dietary supplements. In the following sections, the existing in vitro and in vivo evidence on the biological properties of compounds contained in *Tetraselmis* and *Nannochloropsis* sp. microalgae will be described.

### 3.1. Antioxidant Activity of Microalgae and Derived Compounds

Reactive oxygen species (ROS) and reactive nitrogen species (RNS) are produced under physiological conditions, also introduced into the body by environment pollution, unhealthy dietetic habits, smoking, lack of sleep, and irradiation. At low concentration, ROS and RNS play a vital role on cellular redox signaling and immune function. However, when their concentration increases or the antioxidant defenses decrease, the balance between them is broken, resulting in a state of oxidative stress. In this state, ROS and RNS provoke the oxidation of protein, lipid, and DNA leading to lipid membrane peroxidation, mitochondrial swelling and lysis, mutagenic actions, post-translational protein modifications, and finally cell death [73,74]. Thus, oxidative stress and free radicals are linked to the etiology and pathogenesis of multiple chronic diseases such as neurodegenerative and cardiovascular disorders, obesity, diabetes, several types of cancer, and accelerated aging [75].

The human body has an effective antioxidant defense system composed by enzymatic and non-enzymatic mechanisms. Still, these mechanisms are not complete, and to maintain the oxidative balance, additional antioxidants must be ingested through the daily diet. Thus, the research and consumption of natural antioxidants to prevent damage caused by oxidative stress and reduce the development of various chronic diseases has notably increased in the last years. These natural antioxidants are also attracting interest as natural preservatives to prevent oxidation and spoilage during food manufacture and storage [76]. Although most natural antioxidants currently available on the market are derived from terrestrial plants, microalgae are nowadays being considered as a potential source of natural antioxidant compounds by the food, cosmetic and nutraceutical industries [77].

In Table 7, the existing evidence on the antioxidant activity demonstrated for *Tetraselmis* and *Nannochloropsis* sp. microalgae extracts is summarized. Most studies reporting antioxidant activity have been conducted under in vitro conditions. The studies are mainly based on the radical [2,2′-azino-bis(3-ethylbenzothiazoline-6-sulfonic acid), ABTS], 2,2-diphenyl-1-picrylhydrazyl (DPPH), peroxyl, and hydroxyl scavenging capacity of microalgae extracts, although other mechanisms of action such as iron and copper chelating, ferric reducing antioxidant power (FRAP), and inhibition of lipid peroxidation have also been demonstrated. In general, the results are very heterogeneous depending on the species and strains studied, the antioxidant activity assays, the extraction conditions and the concentrations used. Moreover, the variety in the biochemical composition and the capacity of microalgae to produce a broad range of antioxidant compounds is the basis for the diversity in their antiradical activity. Although few associations have been made between the antioxidant effects of microalgae and the metabolites responsible for the observed effects, some correlations have been detected with phenolic compounds including flavonoids, carotenoids, PUFAs and protein/peptide fractions [77]. As in plants, phenolic compounds are one of the main components responsible for the antioxidant defense mechanisms in microalgae. These compounds mainly act to neutralize ROS through hydrogen atom transfer, although other mechanisms have been described, such as inactivation of radicals by monoelectronic transfer and chelation of transition metals involved in the Fenton reaction, thus preventing the formation of highly reactive hydroxyl radicals [78]. 

Total phenolic compounds present in extracts obtained from *Tetraselmis* sp., *T. suecica* and *N. oculata* with ethanol/water or hexane/ethyl acetate/hot water mixtures were demonstrated to scavenge ABTS radicals, inhibit lipid peroxidation and show FRAP [65]. These compounds have also been reported to mostly contribute to the DPPH radical scavenging capacity of *Tetraselmis* sp. KCTC12236BP extracts [82], the DPPH radical scavenging and iron and cupper chelating capacities of *Nannochloropsis* sp. SBL1 and SBL4 methanolic extracts [98], and the DPPH and NO radical scavenging activity, metal chelating ability, and FRAP values of *N. oculata* extracts [102]. In a recent work, phenolic compounds are recognized as the major factor responsible for the ABTS and DPPH radical scavenging activity and the FRAP values of extracts obtained from *T. marina* IMA043 strain, although the authors suggest that the remaining activity that cannot be explained by the presence of phenols could be ascribed to other molecules present in the extract [85]. Similar findings had been reported in previous studies that recognized both phenolic compounds and PUFAs as the contributors on the antioxidant activity exerted by extracts from *N. oculata* [69], *Tetraselmis* sp. [79], *T. chuii* and *N. granulata* [28]. The correlation between PUFAs content and antioxidant activity of microalgae has been recently reported [76]. These authors determined that species (*C. vulgaris*, *Chlorococcum amblystomatis*, *Scenedesmus obliquus*, and *Phaeodactylum tricornutum*) with higher abundance of PUFAs than *N. oceanica* showed higher DPPH and ABTS radical scavenging activity, while neither omega-3/omega-6 ratio nor MUFAs content exerted any influence. Banskota et al. [28] also suggested that the higher the unsaturation level (especially PUFA content), the better the lipophilic (L)-ORAC values of microalgae extracts. Thus, *T. chuii* had the lowest PUFAs and MUFAs content, and also low L-ORAC values. However, the relationship between microalgae lipids and antioxidant activity needs to be completely elucidated.

Carotenoids have been used as feed for aquaculture and food colorants, becoming very popular as dietary supplements in the last years. They are potent antioxidants for human health and are involved in the modulation of body functions such as cellular signaling and gene expression [107]. Their ability to react with free radicals through hydrogen atom transfer, monoelectronic transfer and adduct formation is already well known [108]. Moreover, due to their conjugated double bond system, carotenoids play an important role in quenching ROS released during photosynthesis, especially singlet oxygen [65]. The carotenoids-rich extracts obtained from different microalgae species have demonstrated to exert potent antioxidant effects through multiple mechanisms. Therefore, the ethanol/water extract from *T. suecica*, containing high levels of carotenoids, showed marked and dose-dependent DPPH radical scavenging activity, comparable to that shown by known antioxidants such as α -tocopherol and ascorbic acid [88]. In this study, the protective effects of the extract were also confirmed in H_2_O_2_-induced lung A549 cells, finding that it could revert the cell viability inhibitory effects of the chemical oxidant, reaching up to 100% recovery at the highest extract dose. Moreover, the extract targeted the expression of dehydrocholesterol reductase-24 (DHCR24) and prostaglandin reductase 2 (PTGR2) genes and proteins, and reduced the levels of prostaglandin E2 (PGE2) [88]. The study by Feller and coworkers [103] also reported that carotenoid compounds were directly correlated with the DPPH radical scavenging capacity of *N. oculata* extracts obtained by both supercritical CO_2_ and subcritical n-butane extraction methods, independent of the extraction method tested. One of the major carotenoids produced by this microalgae specie is zeaxanthin; thus, future studies focusing on demonstrating its contribution on the antioxidant effects of extracts as well as on optimizing the extraction conditions to reach higher carotenoids yield should be of interest. The total carotenoid and lutein contents were also strongly correlated with the ORAC values measured in extracts obtained from different microalgae species including *Tetraselmis* sp., while no correlation was detected for the β-carotene [77]. Moreover, other compounds present in the extracts such as phenolic compounds and fatty acids were suggested to contribute to the lipid peroxidation inhibition shown by the microalgae species. Lutein is a fat-soluble carotenoid, known by its health benefits as antioxidant, anti-inflammatory and protective agent against cardiovascular and Alzheimer’s diseases [109]. In a recent study, the solvent extraction conditions were optimized by response surface methodology (RSM) to improve lutein recovery from microalga *T. suecica* [86]. At the optimized conditions, the lutein content was more than 3-fold higher than that of the control group (before optimization), and the extract showed higher ABTS radical scavenging activity.

In the last years, other compounds present in microalgae have been determined to contribute to the health benefits, namely it was discovered that microalgae proteins can exert biological activities through peptides included into their sequence and released by use of exogenous enzymes from microbial or mammalian origin as well as via gastrointestinal proteases [14]. Bioactive peptides have been determined to exert multiple bioactivities such as antioxidant, anti-microbial, anti-hypertensive, chemopreventive, and modulatory of immune, nervous and/or metabolic systems. However, up to date, few data are available on the production of these bioactive peptides from *Tetraselmis* or *Nannochloropis* proteins. Medina et al. [100] reported that the *N. gaditana* microalgal biomass hydrolyzates with papain showed potent antioxidant activity measured by the ORAC assay. Norzagaray-Valenzuela et al. [90] analyzed the antioxidant capacity of microalgae residual biomass and protein hydrolyzates with alcalase of three green microalgae species: *T. suecica* TES2, *Nannochloropsis* sp. NNX1, and *Dunaliella tertiolecta*. The hydrolyzates showed higher ORAC, DPPH, and ABTS radical scavenging activities than their biomass at the assayed concentrations. Moreover, hydrolyzates from *Nannochloropsis* sp. showed anti-aging effects through inhibition of elastase and hyaluronidase. Although limited, these preliminary findings suggest the role of microalgae proteins as source of multifunctional peptides with promising applications in food, pharmaceutical, and cosmetical industries.

Multiple biological effects such as antioxidant, immunomodulatory antimicrobial, and antitumoral have been ascribed to polysaccharides [110]. Regarding the antioxidant activity, microalgae polysaccharides are known free radical scavengers, and therefore have antioxidant effects preventing oxidative damage in living organisms. Kashif et al. [81] purified water-soluble polysaccharides from defatted microalgal biomass of *Tetraselmis* sp. KCTC 12236BP and KCTC 12432BP, finding a strong correlation between these compounds and the antioxidant, antifungal, and tyrosinase inhibitory activities. More recently, the adaptation of autotrophic to heterotrophic culture was carried out with *T. suecica* (Kylin) Butcher to increase the production of exopolysaccharides and their ABTS radical scavenging capacity and cytotoxic effects against human leukemia, breast, and lung cancer cells [91].

Even though cell models provide more physiological information by considering the bioavailability and metabolism of tested compounds, the available data about the antioxidant activity of microalgae extracts using cellular assays are still scarce (Table 7). In the study of Lauritano et al. [89], the antioxidant activity of the crude extracts of 32 microalgal species including *T. suecica* was evaluated using two different assays in hepatocellular liver carcinoma HepG2 cells: the cellular lipid peroxidation antioxidant activity and the cellular antioxidant activity assays. In addition, the anti-inflammatory, antibacterial, anticancer, and antidiabetic properties of the extracts were evaluated, concluding that the production of primary and secondary bioactive metabolites in microalgae is influenced by the species and strain and the culture media and conditions, growth phases, and clones. This metabolic flexibility favors the discovery of novel bioactive compounds for the management/control of human diseases. Khatib et al. [94] examined the antiatherogenic effects of *Nannochloropsis* sp. extracts, reporting that the 70% ethanol–water extract inhibited macrophages J-774A and LDL oxidation, increased protein paraoxonase 1 (PON1) activity, and protected it from the deleterious effects of linoleic acid hydroperoxide. The authors isolated and purified the compound responsible for the observed effects, the structure of which was elucidated as the lyso-diacylglyceryltrimethylhomoserine lipid.

As it has been described for cellular assays, the animal models reported to evaluate the antioxidant activity of microalgae extracts are still limited in comparison to in vitro assays. In the case of *Tetraselmis* sp. and *Nannochloropsis* sp., few studies can be found in the literature, and they are mainly focused on evaluating the impact of microalgae meal on the antioxidant status of shrimp or fish, suggesting their potential as an alternative to fishmeal in aquafeed [111,112]. Recently, male streptozotocin-induced diabetic rats were fed control either diet supplemented with 10% *N. gaditana* or not, for 2 months. The administration of microalgae was effective in attenuating oxidative stress and inflammation in diabetic rats, reducing glucose and glycated hemoglobin levels, and improving the renal and hepatic functions [113]. Thus, although the number of studies is still scarce, it seems that the inclusion of microalgae directly into the diet could exert benefits on the animal physiology, indicating the promising potential of their use in human or animal nutrition as functional ingredients. However, the digestibility of the microalgae and bioavailability of the antioxidant compounds into the food/supplement matrices are research areas that need to be explored.

### 3.2. Antimicrobial Activity of Tetraselmis and Nannochloropsis Microalgae

In the last years, the demand for new and effective antimicrobial compounds has notably increased due to the evolution of microbial pathogens and the consequent antibiotic resistance. Among the natural sources of antimicrobials, microalgae are being considered one of the most promising because of their capacity to produce numerous and diverse bioactive metabolites that allow them to defend themselves against pathogenic bacteria, viruses, and fungi in the aquatic environment [114]. The antimicrobial activity of different microalgae extracts has been mainly associated to fatty acids [115], phenolic compounds [116], and pigments [117]. Maadane et al. [118] evaluated the antimicrobial effects of the ethanolic extracts obtained from nine microalgae species including *N. gaditana* and *Tetraselmis* sp. against bacteria *Escherichia coli*, *Pseudomonas aeruginosa* and *Staphylococcus aureus*, the yeast *Candida albicans* and the fungus *Aspergillus niger*. The authors linked the observed antimicrobial effects to the contents of the extracts in fatty acids, carotenoids, and phenolic compounds. In the recent study of Wali et al. [106], the extracts obtained from *N. oculata* by using methanol under sonication showed potent inhibitory activity against bacteria *S. aureus* ATCC 25923, *P. aeruginosa* ATCC 27853, and *E. coli* ATCC 25922, and moderate effects against the yeast *C. albicans* ATCC 24433. The extracts also showed potent antioxidant and anticancer properties on breast cancer MDA-MB-231 cells. The terpenoids, carotenoids, polyphenolic, and fatty acids contained in the extracts were suggested as the major factor responsible for the observed effects. More recently, the ethanolic extracts of *N. oceanica* were determined to exert inhibitory effects against *Vibrio harveyi* in an in vitro co-culture assay, although the responsible compounds were not identified [119]. The growth inhibitory effects on *Vibrio anguillariim*, and *Vibrio salmonicida* reported for extracts obtained from *T. suecica* were not associated with any specific compound [120]. Vibriosis is associated with fish infections and death, resulting in enormous economic loss for farmers. Thus, the antibacterial properties of microalgae might be exploited as an alternative for disease prevention and management in the aquaculture industry [121].

In addition to phenolic compounds and fatty acids, peptides and polysaccharides have also been determined to contribute on the antimicrobial activity demonstrated for microalgae extracts. Three peptide sequences identified in the acid extracts of *T. suecica* were determined to exert potent antimicrobial activity against both Gram-positive and Gram-negative bacterial strains [122]. In this work, substitutions of leucine, tyrosine, threonine, and methionine by lysine resulted in a notable increase in the bioactivity, particularly against Gram-positive bacteria. This could be due to the increase in the peptide’s positive net charge, correlated with the lysine side chain, which is able to interact with the negatively charged bacterial membranes [123]. Water-soluble polysaccharides extracted from *N. oculata* and *Tetraselmis* sp. were determined to inhibit the growth of both bacteria and yeasts [81,104]. Although the mechanisms involved in their antimicrobial activity have not been completely elucidated, the capacity of polysaccharides is well known to impact the cytoplasm permeability and to participate in the DNA decomposition after polysaccharide/DNA binding, as well as affect the denaturation of essential bacterial proteins [124]. Therefore, the presence of different antimicrobial compounds in microalgae and the potential synergistic action between them opens the door to new applications of these organisms in aquaculture as well as in the nutritional and human medical fields.

### 3.3. Anti-Carcinogenic Effects of Tetraselmis and Nannochloropsis sp. Microalgae Compounds

Cancer has become one of the world’s leading causes of disease and death with important social and economic repercussions. Thus, the current interest is focused on the search of novel sources of bioactive compounds able to act on carcinogenesis-associated biomarkers and pathways. Among them, natural compounds obtained from various sources have been demonstrated to exert anticancer potential, therefore being promising candidates for the development of new drugs. As a result, currently, the percentage of drugs derived from natural sources corresponds to approximately 60% [125].

Several research studies reported microalgae species including *Tetraselmis* sp. and *Nannochloropsis* sp. with anti-cancer properties (Table 8). Some of these works evaluated the cytotoxic effects of a single application of microalgal extracts. Chloroform extracts obtained from *T. suecica* reduced the viability of breast cancer MCF-7 and 4T1 cells [126]. Moreover, potentiation of the cytotoxic effects and induction of apoptosis were reported when microalga extracts were co-applied with silver nanoparticles [105] or tamoxifen [127]. Methanolic extracts from *N. oculata* were also determined to inhibit breast cancer MDA-MB-231 cell viability in a dose- and time-dependent manner in addition to exerting antioxidant and antimicrobial properties [106]. Different compounds present in microalgae extracts have been described as major contributors to the anticancer properties. Thus, a sterol-rich fraction of cultured marine microalga *N. oculata* showed marked cytotoxic and apoptosis inducing effects on leukemia HL-60 cells, and anti-inflammatory activities against lipopolysaccharide-stimulated RAW 264.7 macrophages [128]. Lipids, mainly omega-3 PUFAs, were determined to be the main factor responsible for the cytotoxic effects against colon cancer HCT-116 cells of *N. gaditana* extracts obtained by an approach combining enzyme and ultrasounds [129]. A water-soluble polysaccharide-enriched extract from *N. oculata* was reported to inhibit proliferation of HeLa cells in a dose-dependent manner, in addition to being antimicrobial and anticholinesterasic [104].

Multifunctionality was also reported for exopolysaccharides purified from the heterotrophic culture of *T. suecica* (Kylin) Butcher that were determined to exert ABTS radical scavenging activity and cell viability inhibitory properties against leukemia, breast, and lung cancer cells [91]. The multifunctionality could represent an additional value for these microalgal products. Proteins have also been described as contributors to the cytotoxic effects of microalgae extracts. Thus, among 488 proteins identified by proteomic analysis of *N. gaditana,* Carrasco-Reinado et al. [130] selected the UCA01 protein belonging to the prohibitin family to evaluate its bioactivity. This protein was demonstrated to exert potent antiproliferative activity against colon adenocarcinoma Caco-2 and hepatocellular carcinoma HepG2 cells, without affecting nontumorigenic cells. Therefore, the recent findings on the anticancer properties of *Tetraselmis* sp. and *Nannochloropsis* sp. microalgae open new possibilities to produce bioactive ingredients with potential health benefits and applications in the food and nutraceutical industry.

## 4. Digestibility of *Tetraselmis* sp. and *Nannochloropsis* sp. Microalgae Biomass

In vitro models that simulate human gastrointestinal digestion provide valuable information on the bioavailability of potentially bioactive compounds present in microalgae biomass. In general terms, the disruption of the cell wall of microalgae prior to digestion is highly recommended to facilitate the action of digestive enzymes. This should be considered especially for microalgae with thick and rigid cell walls, such as *Nannochloropsis* sp., as their digestibility is affected by the selected disruption treatment [14].

In vitro crude protein digestibility (IVPD) is often used to assess the digestibility of microalgae biomass and to determine its potential as a protein source. Wild et al. [22] reported that IVPD was in the range of 48–59% for *N. oceanica* and *N. oculata* when the cell wall was not disrupted, and it was increased to 78–80% when microalgae biomass was treated with a stirred ball mill [22]. Lipid-extracted *N. granulata* and *T. chuii* showed significantly higher levels of IVPD (˃85%) when compared to the same species of whole microalgae [48]. On the other hand, in vitro lipid digestibility (IVLD) is determined by the comparison of free fatty acid concentration released after digestion with the total fatty acid content of the original sample. In this line, lipid digestion of *Nannochloropsis* sp. was considered incomplete in untreated biomass, accounting for 36–40% of IVLD. The application of high pressure homogenization in these microalgae suspensions substantially modified IVLD (56–62%) and it also increased the bioaccessibility of omega-3 PUFAs and carotenoids during the small intestinal phase [132]. Recent research comparing different species of microalgae reported lower IVLD values for *N. gaditana* treated by bead milling (33.3%), suggesting that such pretreatment was not sufficient for effective cell disruption [27].

In terms of in vitro carbohydrate digestibility (IVCD) in different species of microalgae, their values tend to mimic those of organic matter digestibility except in microalgae with high ash content (˃20%) [30]. Free glucose is the main product of IVCD, and its release depends on microalgae characteristics. Verspreet et al. [27] observed that disrupted *N. gaditana* only yielded 1.8 g of free glucose/100 g of dry matter, while other species produced 10 times more. They related this fact to the β-linkages of glucose units that would not be susceptible to degradation by human α-amylases or disaccharidases [27]. In addition, it is also important to consider that culture conditions can greatly affect the carbohydrate content of microalgae. For instance, *T. suecica* presents an average carbohydrate content of approximately 10% when it is grown in a medium that provides all the nutrients, and it can reach 37% when growing medium is deprived of a nitrogen source. However, it appears that those changes in carbohydrate composition would have little effect on IVCD and organic matter digestibility [30].

In vivo investigations designed to understand the human digestion process of microalgae biomass are very scarce and further research is needed. Functional extracts obtained from untreated and treated *N. gaditana*, through a combined thermal and high-pressure treatment, were recently evaluated in vivo using a rat model with a 20% dietary inclusion level of microalgae [99]. Although both untreated and treated microalgae showed interesting potential for dry matter digestibility and antioxidant capacity (i.e., polyphenol content, ABTS and inhibition of lipid peroxidation), treated *N. gaditana* was able to improve all biomarkers. The in vivo apparent digestibility coefficient accounted for 67% and 83% in rats fed the untreated and treated microalgae biomass, respectively. In addition, the fecal microstructure of rats fed untreated *N. gaditana* clearly showed undigested microalgae and their microbiota was different compared to that of the rats fed the treated microalgae. It would suggest that the technological treatment not only favors the release of certain metabolites but could also promote the growth of specific microbiota [99]. Overall, we can conclude that microalgae biomass processing leading to cell disruption is a key factor for nutrient release and consequent digestibility of the compounds of interest.

## 5. Digestibility of *Tetraselmis* sp. and *Nannochloropsis* sp. in Food Products

*T. chuii* dried biomass has been authorized in the EU for commercialization as novel food and food supplement [7,133]. The intake of this microalgae is safe, and its unique nutritional composition makes it a promising sustainable ingredient for the agrifood industry [134,135]. *T. chuii* has been used to replace wheat flour in the manufacture of protein-enriched bread [20] and it has also been incorporated in buckwheat, rice flour and potato starch formulations to increase the bioactivity of gluten-free bread [136]. Qazi et al. [137] evaluated the IVPD of raw and ethanol-treated *T. chuii* biomass, either alone or incorporated into wheat bread, following the standardized INFOGEST static in vitro digestion model. Breads including 12% of disrupted *T. chuii* showed significantly higher enzymatic degradation of proteins into small peptides than bread without microalgae. Dissolved protein represented 83% in the commodity wheat flour bread, and it was reduced to 59–67% by incorporating microalgae biomass. As a result, IVPD decreased in breads formulated with *T. chuii*, especially when microalgae biomass was treated with ethanol [137].

In vitro digestibility of *T. suecica* in food products has also been evaluated. Crackers formulated with 2 and 6% of untreated microalgae biomass showed a similar dry matter digestibility to crackers without microalgae (~80–85%). IVPD values ranged from 50 to 65% in *T. suecica* crackers, slightly lower than the 75% IVPD of commercial crackers, but the level of added microalgae biomass did not affect either dry matter digestibility or IVPD [138]. Similar contents of *T. suecica* were also incorporated into cookies when wheat flour was replaced by 2 and 6% of untreated microalgae biomass. No changes were observed when comparing the in vitro digestibility of cookies with distinct species of microalgae at different concentrations. However, prior to cookies manufacture, the evaluation of the in vitro digestibility revealed differences between the microalgae species [139]. This would highlight the importance of the food matrix in in vitro digestive simulations and thus, further research is encouraged to fully understand the digestibility of microalgae in complex food products. On the other hand, to our knowledge, there is no published research evaluating the digestibility of *Nannochloropsis* sp. incorporated into a food matrix. Although there is a huge commercial interest in this microalga as a food ingredient, it has not yet been authorized as novel food due to the difficulty of complying with all strict safety criteria imposed by the current legislation [17]. In the coming years, an increase in patent applications and commercial authorizations related to *Nannochloropsis* sp. is expected, but it is essential to evaluate the digestibility of these microalgae-enriched food products before their launch on the market.

## 6. Conclusions

*Tetraselmis* sp. and *Nannochloropsis* sp. are excellent sources of a diversity of bioactive compounds, including lipids, proteins, carbohydrates, minerals, and vitamins. Their variable composition depends to a large extent on the growing and drying conditions of the microalgae biomass, which substantially affects their bioactivity and digestibility. Most of the research on bioactivity has been focused on determining the antioxidant capacity of *Tetraselmis* and *Nannochloropsis* sp. extracts under different pretreatment and extraction conditions. Research on other potential bioactivities (i.e., antibacterial, antifungal, anti-inflammatory, anti-diabetic, anti-obesity, anti-aging, anti-cancer) is expected to increase in the coming years to define the key compounds responsible of such bioactivities and design microalgae culture conditions that allow to maximize their potential. Regarding the digestibility of *Tetraselmis* and *Nannochloropsis* microalgae, the available information is still scarce. Further investigations are encouraged to assess the potential impact that the food matrix could exert on the behavior of the microalgae under physiological conditions to select the most suitable form of microalgae administration.

## Figures and Tables

**Table 1:** Amino acid composition (expressed as g/16 g of nitrogen) of *Nannochloropsis* sp. and *Tetraselmis* sp. microalgae and comparison with recommended values by the Food and Agriculture Organization (FAO).

Amino Acid	Content (g/16 g Nitrogen)					
	FAO	*Nannochloropsis* ^1^	*N. granulata* ^2^	*N. oculata* ^2^	*T. chuii* ^2^	*T. suecica* ^2^
Essential						
Isoleucine	4.0	3.6–4.2	5.6	4.8	3.5	3.5
Leucine	7.0	7.9–8.4	11.0	7.8	7.5	8.0
Valine	5.0	4.8–5.4	7.1	6.5	5.8	5.7
Lysine	5.5	5.2–6.0	8.5	6.1	5.7	6.0
Phenylalanine	6.0	4.4–4.8	6.2	6.2	5.4	5.9
Methionine	3.5	1.7–2.1	3.5	1.6	1.9	2.3
Tryptophan	1.0	1.5–1.8	2.8	1.6	3.7	3.8
Threonine	4.0	4.5–4.9	5.4	5.5	4.2	4.1
Non-essential						
Tyrosine		2.8–3.2	4.2	4.2	3.7	3.8
Cysteine		0.6–0.8	1.6	0.4	0.6	0.7
Alanine		6.2–6.9	7.1	7.4	6.8	6.9
Arginine		5.1–5.4	7.4	7.3	13.5	13.2
Aspartic acid		8.2–8.6	11.4	7.6	9.4	8.9
Glutamic acid		9.9–12.0	14.1	10.1	12.4	11.2
Glycine		5.1–5.4	7.5	5.5	5.9	5.9
Histidine		2.0–2.1	2.3	2.1	1.8	1.8
Proline		4.3–7.8	11.2	9.3	5.1	4.7
Serine		4.2–4.3	5.6	5.0	4.3	4.6
TAA		82.0–94.1	122.5	99.0	101.2	101.0
HAA		37.8–45.4	60.3	49.8	44.0	45.3
AAA		8.7–9.8	13.2	12.0	12.8	13.5

^1^ Values from Wild et al. [22]; ^2^ values from Kumar et al. [14]. AAA: aromatic amino acids (phenylalanine, tryptophan, and tyrosine); HAA: hydrophobic amino acids (alanine, valine, isoleucine, leucine, tyrosine, phenylalanine, tryptophan, methionine, proline, and cysteine); TAA: total amino acids.

**Table 2:** Composition of fatty acids (expressed as mg/g of dry weight, DW) of *Nannochloropis* sp. and *Tetraselmis* sp. microalgae biomass.

Fatty Acid	Fatty Acids (mg/g DW)									
	*N. oculata* ^1^	*N. limnetica* ^2^	*N. gaditana* ^3^	*N. granulata* ^4^	*N. oceanica* ^5^	*N. oceanica* ^6^	*T.* sp. ^5^	*T. suecica* ^6^	*T. chuii* ^4^	*T. chuii* ^7^
**SFA**										
12:0	---	---	0.4	1.4	1.2	3.5	---	---	---	0.02
14:0	2.7	6.3	4.1	11.3	16.9	9.3	0.5	---	---	0.2
16:0	14.6	36.1	17.9	27.7	17.2	67.3	6.3	13.3	13.9	14.9
18:0		0.7	0.3	---	1.8	3.5	1.2	---	---	0.4
MUFA										
16:1	21.8	37.1	31.5	41.5	18.2	58.2	1.3	0.4	---	0.4
*cis*-9 18:1	6.3	30.1	1.9	11	4.1	30.6	10.7	19.5	6	18.4
20:1	---	---	---	---	0.5	---	0.9	---	1.2	1.7
PUFA										
16:2 n-4	---	---	---	---	---	---	---	---	1.8	---
18:2 n-6	2.4	3.8	1.4	7.7	9.7	2.8	2.5	11.9	6.2	4.8
18:3 n-6	---	0.2	0.8	---	---	0.7	---	---	---	0.5
20:2 n-6	---	---	---	---	0.5	0.4	---	---	---	0.04
20:4 n-6	5.8	5.3	3.2	---	3.7	4.8	0.6	---	---	0.4
18:3 n-3	---	0.4	0.1	---	0.5	0.2	6.4	17.6	10.8	9.7
18:4 n-3	---	---	---	---	---	---	4.1	---	---	---
20:5 n-3	23.3	28.1	54.9	31.7	23.4	31.5	4.8	---	3.8	4.1
22:6 n-3	---	0.1	0.20	---	---	---	0.2	---	---	---

^1^ Volkman et al. [25]; ^2^ Krienitz and Wirtz [26]; ^3^ Verspreet et al. [27]; ^4^ Banskota et al. [28]; ^5^ Patil et al. [29]; ^6^ Niccolai et al. [30]; ^7^ Sandgruber et al. [21]. MUFA: monounsaturated fatty acids; PUFA: polyunsaturated fatty acids; SFA: saturated fatty acids.

**Table 3:** Minerals (major and trace elements, expressed as mg/100 g of dry weight, DW) composition of *Nannochloropsis* sp. and *Tetraselmis* sp. microalgae.

Mineral	Content (mg/100 g DW)							
	*N.* sp. ^1^	*N.* sp. ^2^	*N. granulata* ^3^	*N. gaditana* ^4^	*T. chuii* ^3^	*T. chuii* ^4^	*T.* CTP4 ^5^	*T. suecica* ^6^
Major elements								
Calcium	972	590	90	631	2990	185	1190	1791
Magnesium	316	530	260	370	430	305	2080	601
Phosphorus		1300	730	887	1460		710	964
Potassium	533	1500	1500	1261	1860		4200	1349
Sodium	659	2500	1030	2022	890		1180	3223
Sulphur		610	580		1380			
Trace elements								
Copper	35	1.5	1. 8	2.9	10.2	0.5	1.1	1.1
Iron	136	62.7	139.5	48.9	177.4	51.8	32.3	30.9
Iodine		0.2				0.3	0.1	
Manganese	3.4	6.9	15.1	9.3	19.1	0.1		3.5
Selenium		0.01	0.1		0.1			
Zinc	103	5.5	3.2	10.1	6.4	2.1	2.9	6.6
Nickel						0.01		
Molybdenum						0.05		

^1^ Rebolloso-Fuentes et al. [32]; ^2^ Wild et al. [22]; ^3^ Tibbetts et al. [48]; ^4^ Sandgruber et al. [21]; ^5^ Pereira et al. [16]; ^6^ Di Lena et al. [42].

**Table 7:** Mechanisms of antioxidant activity and potential responsible compounds of the observed effects demonstrated for *Tetraselmis* sp. and *Nannochloropsis* sp. microalgae extracts by biochemical assays and cell models.

Microalgae	Pretreatment/Extraction Conditions	Mechanisms	Responsible Compound	Ref.
*Tetraselmis* sp.	(1) Hexane (2) Sequential extraction with ether, acetone and water	DPPH radical scavenging, and iron and copper chelating	PUFAs and phenolic compounds	[79]
	Ethanol and ethanol/water or ultrasound + water	DPPH radical scavenging	---	[64]
	Methanol/dichloromethane + ultrasound or acetone + ultrasound	ORAC radical scavenging, and inhibition of lipid peroxidation (TBARS)	Xanthophylls	[80]
	Ethanol/water or hexane + ethyl acetate + hot water	ABTS radical scavenging, FRAP, and inhibition of linoleic acid oxidation	Phenolic compounds	[65]
	Methanol/dichloromethane + ultrasound	Inhibition of lipid peroxidation (TBARS)	---	[77]
*T.* sp. KCTC12236BP and KCTC12432BP	(1) 1 N H_2_SO_4_, 1 M NaOH and distilled water (2) Ethanol precipitation	ABTS and DPPH radical scavenging and FRAP	Polysaccharides	[81]
*T.* sp. KCTC12236BP	Ultrasound + water:ethanol	DPPH radical scavenging	Total phenolic compounds and epigallocatechin gallate	[82]
*T.* sp. M8	Water, hexane or ethyl acetate	ORAC	Carotenoids	[62]
*T.* sp. IMP3, and *T.* sp. CTP4	Water or ethanol 96%	ABTS and DPPH radical scavenging and FRAP	Phenolic compounds and PUFAs	[66]
*T. chuii*	Methanol or (1) hexane/dichloromethane and (2) acetone/water/acetic acid	ORAC and DPPH radical scavenging	PUFAs and phenolic compounds	[28]
	Dichloromethane (Folch method)	ABTS and DPPH radical scavenging	PUFAs and other compounds	[76]
	(1) Pulsed electric field (2) DMSO or distilled water	ABTS radical scavenging	Polyphenols and pigments	[83]
	Ethanol, ethyl acetate, or water	DPPH radical scavenging, and iron and cupper chelating	---	[84]
	Water, hexane or ethyl acetate	ORAC	Carotenoids	[62]
*T. marina* IMA043	(1) Ultrasound (2) Water, methanol or dichloromethane	DPPH and ABTS radical scavenging, iron/cupper chelating, and FRAP	SFA, MUFAs and phenolic compounds	[85]
*T. suecica*	Distilled water, hexane, heptane, ethyl acetate, acetone, ethanol or methanol	ABTS radical scavenging, and FRAP	Lutein	[86]
	Ultrasound + methanol:water, methanol or ethanol	DPPH and ABTS radical scavenging, and β-carotene bleaching	Multiple compounds	[87]
	Ethanol:water	DPPH radical scavenging	Carotenoids	[88]
	Ethanol/water or hexane + ethyl acetate + hot water	ABTS radical scavenging, FRAP, and inhibition of linoleic acid oxidation	Phenolic compounds	[65]
	Water, hexane or ethyl acetate	ORAC	Carotenoids	[62]
	Water + ultrasound + acetone	Cellular antioxidant and lipid peroxidation inhibition in HepG2 cells	---	[89]
*T. suecica* TES2	(1) Ultrasound + chloroform:methanol or ethanol (2) Enzymatic hydrolysis	ORAC, DPPH, and ABTS radical scavenging	Protein/peptide fractions	[90]
*T. suecica* (Kylin) Butcher	(1) Phenolics removal with polyvinylpyrrolidone (2) Ethanol or N-cetylpyridinium bromide extraction	ABTS and DPPH radical scavenging	Exopolysaccharides	[91]
*T. striata* CTP4	Ethanol, ethyl acetate, or water	DPPH radical scavenging, and iron and cupper chelating	---	[84]
*T. tetrathele*	Methanol	DPPH radical scavenging, and inhibition of lipid and linoleic acid peroxidation	---	[92]
*Nannochloropsis* sp.	Ethyl acetate, methanol, water, isopropanol or petroleum ether	Total capacity, hydroxyl and NO radical scavenging, metal chelating, and FRAP	Fatty acids (hexadecanoic acid)	[93]
	Ethanol:water	Inhibition of LDL oxidation and ROS inhibition in H_2_O_2_-induced macrophages J-774A	Lyso-diacetylglyceryltrimethylhomoserine	[94]
	Methanol	Total capacity; DPPH, superoxide, and hydroxyl radical scavenging	---	[95]
	Ethanol, ethyl acetate, or water	DPPH radical scavenging, and iron and cupper chelating	---	[84]
	Methanol + ultrasound	ABTS and DPPH radical scavenging, FRAP, and ferrous ion-chelating ability	Phenolic compounds and carotenoids	[57]
	Water, hexane or ethyl acetate	ORAC	Carotenoids	[62]
	Hexane, dichloromethane, chloroform or methanol	DPPH, superoxide anion radical scavenging, FRAP, and ferrous-ion chelating	---	[96]
	Ethanol + water	ABTS radical scavenging	---	[97]
*N.* sp. NNX1	(1) Ultrasound + chloroform:methanol or ethanol (2) Enzymatic hydrolysis	ORAC, DPPH, and ABTS radical scavenging	Protein/peptide fractions	[90]
*N.* sp. SBL1 and SBL4	Methanol	DPPH radical scavenging and iron and cupper chelating	Phenolic compounds	[98]
*N. gaditana*	Ethanol and ethanol/water or ultrasound + water	DPPH radical scavenging	---	[64]
	Ethanol:water:12N HCl	ABTS and DPPH radical scavenging, and lipid peroxidation inhibition	---	[99]
	(1) High pressure disruption (2) Alkaline pH and isoelectric protein precipitation (3) Papain hydrolysis	ORAC radical scavenging	Protein/peptide fractions	[100]
	Supercritical CO_2_	DPPH radical scavenging, β-carotene bleaching, and FRAP	Lipids	[101]
*N. granulata*	Methanol or (1) hexane/dichloromethane and (2) acetone/water/acetic acid	ORAC and DPPH radical scavenging	PUFAs and phenolic compounds	[28]
*N. limnetica* 0065NA	Chloroform:methanol	DPPH radical scavenging	Polar lipids	[35]
*N. oceanica*	Dichloromethane (Folch method)	ABTS and DPPH radical scavenging	PUFAs and other compounds	[76]
*N. oceanica* 0011NN	Chloroform:methanol	DPPH radical scavenging	Polar lipids	[35]
*N. oculata*	Ethyl acetate or methanol	DPPH and NO radical scavenging, metal chelating, and FRAP	Phenolic and flavonoid compounds	[102]
	(1) Supercritical CO_2_ (2) Subcritical n-butane extracts	DPPH radical scavenging	Carotenoids	[103]
	(1) Methanol (2) Water	DPPH radical scavenging	Polysaccharides	[104]
	Methanol, ethanol:water, chloroform, or hexane (Soxhlet)	DPPH radical scavenging	Hexanedioic acid, bis (2-ethylhexyl)ester	[105]
	Methanol:water:acetic acid:ascorbic acid + ultrasound or methanol:acetone:hexane or ethyl acetate:hexane + ultrasound or methanol:water:HCl + ultrasound	ABTS and ORAC radical scavenging, and FRAP	Polyphenols, carotenoids, chlorophylls, and triterpenoids	[2]
	Methanol + ultrasound	DPPH, H_2_O_2_, and ABTS radical scavenging	---	[106]
	Ethanol/water or hexane + ethyl acetate + hot water	ABTS radical scavenging, FRAP, and inhibition of linoleic acid oxidation	Phenolic compounds	[65]
	Hexane or methanol	DPPH radical scavenging, and iron and copper chelating	Phenolic compounds and PUFAs	[69]
	Methanol	DPPH radical scavenging, and inhibition of lipid and linoleic acid peroxidation	---	[92]

ABTS: 2,2′-azino-bis(3-ethylbenzothiazoline-6-sulfonic acid); DMSO: dimethyl sulfoxide; DPPH: 2,2-diphenyl-1-picrylhydrazyl; FRAP: ferric reducing antioxidant power; MUFA: monounsaturated fatty acids; NO: nitric oxide; ORAC: oxygen radical absorbance capacity; PUFA: polyunsaturated fatty acids; ROS: reactive oxygen species; SFA: saturated fatty acids; TBARS: thiobarbituric acid reactive substances.

**Table 8:** Biological activities demonstrated for *Tetraselmis* and *Nannochloropsis* sp. microalgae extracts.

Microalgae Specie/Strain	Antimicrobial Activity (Microorganism)	Anti-Carcinogenic Activity (Cell Model)	Other Bioactivities	Ref.
*Tetraselmis* sp.	---	Cell viability inhibition (HepG2)	Acetylcholinesterase inhibition	[79]
*T.* sp. KCTC12236BP and KCTC12432BP	Antifungal (*Candida albicans* and *Penicillium italicum*)	---	Tyrosinase inhibition	[94]
*T. chuii*	---	Cell viability inhibition (HepG2)	Calcium chelating	[84]
*T. suecica*	---	Cell viability inhibition and apoptosis induction (MCF-7 and 4T1)	---	[127]
	Antibacterial (*Escherichia coli*, *Pseudomonas aeruginosa*, and *Staphylococcus aureus*) Antifungal (*Candida albicans* and *Aspergillus niger*)	---	---	[118]
	---	Anti-angiogenic through over-expression of VEGF (PC-3 cells)	---	[87]
	---	Repairing effects through increasing expression of DHCR24 and PTGR1 genes and proteins and reducing PGE2 (A549)	Repairing effects on reconstructed human epidermal tissue cells (EpiDerm^TM^)	[88]
*T. suecica* CCAP904	Antibacterial (*Escherichia coli*, *Salmonella typhimurium*, *Pseudomonas aeruginosa*, *Bacillus cereus*, *Staphylococcus aureus*, *Listeria monocytogenes*, *Micrococcus luteus*)	---	---	[122]
*T. suecica* TES2	---	---	Anti-aging potential through inhibition of elastase and hyaluronidase	[90]
*T. suecica* (Kylin) Butcher	---	Cell viability inhibition (MCF-7, HL-60, and NCI-H460)	---	[91]
*T. striata* CTP4	---	---	Calcium chelating Anti-inflammatory through decreasing TNF-α in LPS-induced macrophages THP-1	[84]
*Nannochloropsis* sp.	---	---	Increase in anti-atherogenic paraoxonase 1 activity	[94]
	---	---	Calcium chelating Anti-inflammatory through decreasing TNF-α in LPS-induced macrophages THP-1	[84]
*N.* sp. NNX1	---	---	Anti-aging potential through inhibition of elastase and hyaluronidase	[90]
*N. gaditana*	---	Cell viability inhibition (Caco-2 and HepG2)	---	[130]
	---	Cell viability inhibition (HCT-116)	---	[129]
	---	---	Skin protection properties by mediating oxidative responses and apoptosis (H_2_O_2_-challenged dermal fibroblasts)	[131]
*N. oceanica*	Antibacterial (*Vibrio harveyi*)	---	---	[119]
*N. oculata*	Antibacterial (*Enterococcus faecalis*, *Staphylococcus aureus*, *Escherichia coli*, and *Pseudomonas aeruginosa*) Antifungal (*Candida albicans*, *Candida glabrata*, *Candida kreusei*, and *Candida parapsilosis*)	Cell proliferation inhibition (HeLa)	Anti-cholinesterase activity	[104]
	---	Cell viability inhibition and apoptosis induction (MCF-7 and 4T1)	---	[105]
	---	Cell viability inhibition and apoptosis induction (MCF-7 and 4T1)	---	[127]
	---	Cell viability inhibition and apoptosis induction (HL-60)	Anti-inflammatory through decreasing LPS-induced iNOS and COX-2 protein levels (RAW264.7)	[128]
	---	---	Anti-aging through inhibition of acetylcholinesterase and butyrylcholinesterase Anti-diabetic through inhibition of α-amylase Anti-obesity through inhibition of pancreatic lipase	[2]
	Antibacterial (*Staphylococcus aureus*, *Pseudomonas aeruginosa*, and *Escherichia coli*) Anti-fungal (*Candida albicans*)	Cell viability inhibition (MDA-MB-231)	---	[106]

COX-2: cyclooxygenase-2; DHCR24: dehydrocholesterol reductase-24; iNOS: inducible nitric oxide synthase; PGE2: prostaglandin E2; PTGR1: prostaglandin reductase 1; TNF-α: tumor necrosis factor-alpha; LPS: lipopolysaccharide; VEGF: vascular endothelial growth factor.

## Data Availability

Not applicable.
